# Microtubule-associated proteins WDL5 and WDL6 play a critical role in pollen tube growth in *Arabidopsis thaliana*

**DOI:** 10.1080/15592324.2023.2281159

**Published:** 2023-11-15

**Authors:** Takashi Okamoto, Hiroyasu Motose, Taku Takahashi

**Affiliations:** Department of Biological Science, Graduate School of Environmental, Life, Natural Science and Technology, Okayama University, Okayama, Japan

**Keywords:** Arabidopsis, pollen germination, pollen tube growth, the WVD2/WDL family

## Abstract

Morphological response of cells to environment involves concerted rearrangements of microtubules and actin microfilaments. A mutant of *WAVE-DAMPENED2-LIKE5* (*WDL5*), which encodes an ethylene-regulated microtubule-associated protein belonging to the WVD2/WDL family in *Arabidopsis thaliana*, shows attenuation in the temporal root growth reduction in response to mechanical stress. We found that a T-DNA knockout of *WDL6*, the closest homolog of *WDL5*, oppositely shows an enhancement of the response. To know the functional relationship between *WDL5* and *WDL6*, we attempted to generate the double mutant by crosses but failed in isolation. Close examination of gametophytes in plants that are homozygous for one and heterozygous for the other revealed that these plants produce pollen grains with a reduced rate of germination and tube growth. Reciprocal cross experiments of these plants with the wild type confirmed that the double mutation is not inherited paternally. These results suggest a critical and cooperative function of WDL5 and WDL6 in pollen tube growth.

While plant cell shape is constrained and maintained by a tough outer cell wall, its dynamics during growth and response to environmental stress involve the coordinated redistribution of microtubules and actin microfilaments.^1^ Organization and function of microtubules are regulated by microtubule-associated proteins (MAPs). The WAVE-DAMPENED2 (WVD2)/WVD2-LIKE (WDL) family in *Arabidopsis thaliana* is a class of plant MAPs, and the members have been shown to play roles in anisotropic cell expansion (WVD2 and WDL1),^2,3^ hypocotyl elongation in response to ethylene (WDL5),^4–6^ brassinosteroids (MDP40)^7^ and light (WDL3 and MDP60),^8–10^ auxin-mediated apical hook opening (WDL4),^11,12^ and stomatal closure in response to ABA (WDL7).^13^ Previously, we have found that the temporal growth reduction of the root exposed to mechanical impedance involves ethylene signaling,^14^ and this response is attenuated in the *wdl5* mutant.^15^ To know the functional relationship between WDL5 and its closest homolog WDL6, which belong to the same clade, WDLB,^16^ we focused here on a knockout mutant of *WDL6*.

T-DNA insertion alleles of *wdl5–2* (GABI-362D09) and *wdl6* (SALK_026362C), named hereafter *wdl6–1* (Figure 1a), were obtained from the Arabidopsis Biological Resource Center. We confirmed no expression of the *WDL6* mRNA encompassing the site of the T-DNA insertion in *wdl6–1* (Figure 1b). Like *wdl5–2*, *wdl6–1* shows the wild-type phenotype under normal growth conditions (Figure 1c, d). We found that, in contrast to *wdl5–2*, *wdl6–1* shows a slight but significant enhancement of the growth reduction of the root in response to mechanical blockage under our experimental system using dialysis membrane-covered agar plates^15^ (Figure 1e). The enhancing effect of *wdl6–1* on the root growth reduction was also detected when the seedling roots were exposed to the agar media containing an ethylene precursor, 1-aminocyclopropane-1-carboxylic acid (ACC)(Figure 1f), suggesting that WDL6 also functions downstream of ethylene but in a different manner from WDL5. We then crossed these mutant alleles but found no individuals of *wdl5–2 wdl6–1* in the F2 generation. Because we could obtain plants that are homozygous for *wdl5–2* and heterozygous for *wdl6–1* and those vice versa, we examined the segregation of genotypes of progeny from self-crosses of these plants but identified no double mutants (Table 1), suggesting that either male or female gamete development is affected by the double mutation. Reciprocal crosses of these plants with the wild-type Columbia (Col-0) indicated that the *wdl5–2 wdl6–1* double mutation can be inherited maternally but not paternally (Table 2). We confirmed that these *wdl5–2 wdl6–1/+* and *wdl5–2/+ wdl6–1* plants have no aborted seeds in siliques and thus examined the rate of pollen germination and the length of pollen tubes *in vitro*. Pollen grains of Col-0, *wdl5–2*, *wdl6–1*, *wdl5–2 wdl6–1/+*, and *wdl5–2/+ wdl6–1* were spread on a dialysis membrane-covered pollen growth medium containing 0.5% agarose, 18% sucrose, 0.01% boric acid, 1 mM CaCl_2_, 1 mM Ca(NO_3_)_2_, 1 mM KCl, 0.03% casein enzymatic hydrolyzate, 0.01% myo-inositol, and 0.01% ferric ammonium citrate, pH 8.0. After incubation for 24 h, the dialysis membrane was moved onto a microscopic slide, and germinating pollens were observed under microscopy as described in previous literature.^17^ Our analysis using ImageJ indicated that the rate of pollen germination and the length of germinating pollen tubes in *wdl5–2* and *wdl6–1* single mutants were reduced compared with those in the wild type (Figure 2). Pollens from *wdl5–2 wdl6–1/+* and *wdl5–2/+ wdl6–1* plants were found to have a further reduced rate of germination (Figure 2a). Furthermore, the length of germinating pollen tubes from *wdl5–2 wdl6–1/+* and *wdl5–2/+ wdl6–1* plants was generally much shorter than that of the wild type and was also significantly shorter than that of *wdl5–2* and *wdl6–1* single mutants (Figure 2b). These results suggest that the pollen germination ability is severely reduced in *wdl5–2 wdl6–1* and, even if *wdl5–2 wdl6–1* pollens could germinate, the delay or decrease in tube growth result in the failure of fertilization.

**Figure 1.**
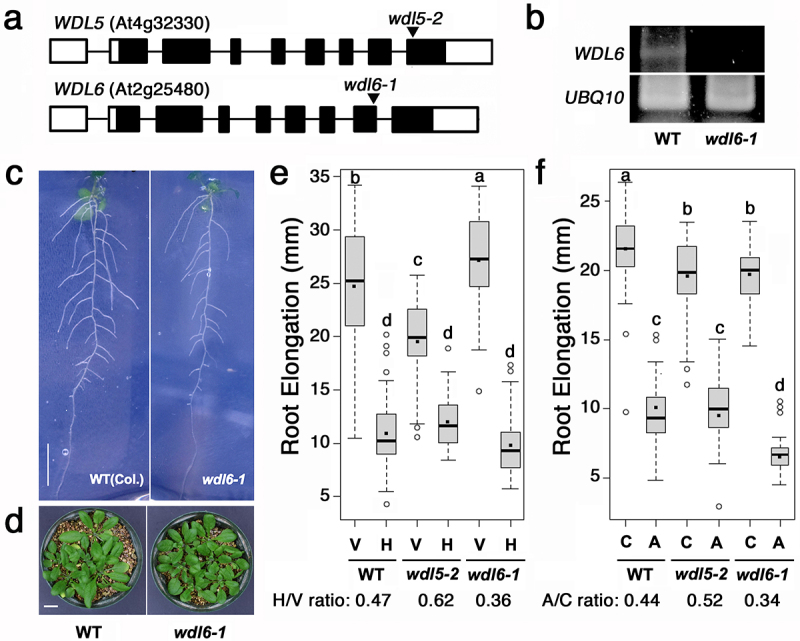
The gross phenotype of *wdl6–1* and the root growth response to mechanical stress in *wdl5–2* and *wdl6–1*. (a) Exon-intron structure of *WDL5* and *WDL6*. Open boxes and filled boxes indicate non-coding and coding exons, respectively. T-DNA insertion sites in *wdl5–2* and *wdl6–1* are shown by arrow heads. (b) Expression of *WDL6* in wild-type and *wdl6–1* seedlings. Total RNA was prepared using PureLink RNA mini kit (Invitrogen), reverse transcribed using the PrimeScript II 1st strand cDNA synthesis kit (Takara) with oligo(dT) primer, and subjected to PCR of 40 cycles using gene-specific primers, WDL6-F (ATGGA CTCTG AAAGC GTCGT), WDL6-R (TTAAG GCTCA ACCGC AACCA), UBQ10-F (GACCA TAACC CTTGA GGTTG AATC), and UBQ10-R (AGAGA GAAAG AGAAG GATCG ATC). Amplified products were detected by agarose gel electrophoresis. (c) phenotype of wild-type and *wdl6–1* seedlings grown for 10 d on MS agar plates. Bar = 1 cm. (d) phenotype of wild-type and *wdl6–1* plants grown for 28 d on vermiculite. Bar = 1 cm. (e) net root growth for 2 d after transfer of 2-d-old seedlings grown on vertical plates to vertical (V) or horizontal (H) plates covered by a dialysis membrane.^15^ (f) net root growth for 2 d after transfer of 2-d-old seedlings grown on vertical plates to plates without (C) or with 100 nM ACC (A).^15^ in (e) and (f), over 30 samples were measured per boxplot. The horizontal bar in the box indicates the median and the black dot indicates the average. The upper and lower hinges of the box indicate 75% and 25% ranges of values, respectively. The upper and lower extreme bars of the box plot indicate the maximum and minimum values, respectively. Different letters indicate statistically significant differences according to one-way ANOVA with Tukey–Kramer multiple comparison test (*P* < .05). All statistical analyses were performed using R (the R Foundation for statistical Computing, Vienna, Austria).

**Figure 2.**
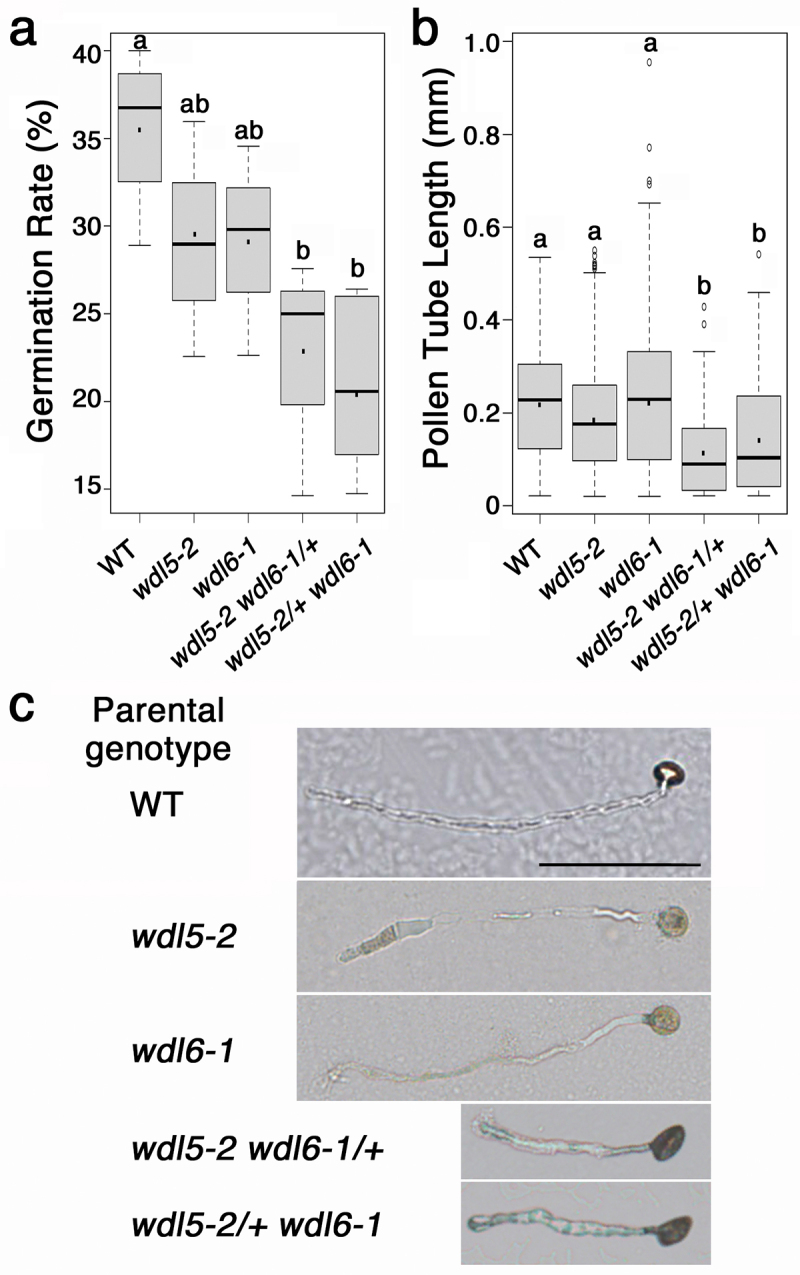
Effect of *wdl5–2* and *wdl6–1* on pollen growth. (a) Germination rate of pollens derived from anthers of indicated genotypes. *In vitro* experiments of pollen germination and tube growth were performed as described previously.^17^ Fifty pollen grains per plate were randomly chosen to check if they were germinated or not and the experiment was repeated five times. (b) Length of pollen tubes derived from anthers of indicated genotypes. Fifty pollen tubes per plate were randomly chosen to measure their length and the experiment was repeated five times. The horizontal bar in the box indicates the median and the black dot indicates the average. The upper and lower hinges of the box indicate 75% and 25% ranges of values, respectively. The upper and lower extreme bars of the box plot indicate the maximum and minimum values, respectively. In (a) and (b), different letters indicate statistically significant differences by one-way ANOVA with Tukey–Kramer multiple comparison test (*P* < .05). All statistical analyses were performed using R (the R Foundation for statistical Computing, Vienna, Austria). (c) Representative phenotypes of pollens grown *in vitro* for 24 h after being extracted from anthers of indicated genotypes. Bar = 100 μm.

**Table 1.** Segregation of *wdl5–2* and *wdl6–1* alleles in self-crossed plants.

Parental genotype	Genotype of F1 plants				
	*wdl5–2 wdl6–1*	*wdl5–2 wdl6–1/+*	*wdl5–2 WDL6*	*wdl5–2/+wdl6–1*	*WDL5 wdl6–1*
*wdl5–2 wdl6–1/+*	0	28	20	-	-
*wdl5–2/+ wdl6–1*	0	-	-	25	23

**Table 2.** Segregation of *wdl5–2* and *wdl6–1* alleles in plants crossed with the wild type.

Parental genotype	Genotype of F1 plants		
(female x male)	*wdl5–2/+ wdl6–1/+*	*wdl5–2/+ WDL6*	*WDL5 wdl6–1/+*
*wdl5–2 wdl6–1/+* x WT	22	26	-
WT x *wdl5–2 wdl6–1/+*	0	48	-
*wdl5–2/+ wdl6–1* x WT	25	-	23
WT x *wdl5–2/+ wdl6–1*	0	-	48

In conclusion, this study reveals an essential role of either WDL5 or WDL6 in pollen germination and tube growth in terms of competitive fertilization success. Because a moderate reduction in the length of pollen tubes was observed in each single mutant, the pollen phenotype might be attributed to dosage effects of these genes. Notably, however, our results showed an opposite effect of these mutations on the root growth response to mechanical stress. Considering that the T-DNA is inserted in the exon corresponding to the protein sequence relatively close to the C-terminus in both *wdl5–2* and *wdl6–1* (Figure 1a), it is possible that both or either of these two mutants represent weak alleles producing a truncated form of the protein. The region encoding the KLEEK domain, a stretch of approximately 90 amino acids, which is conserved in the WVD2/WDL family of proteins and implicated in the interaction with microtubules,^2,3^ lies upstream of T-DNA insertion sites in *wdl5–2* and *wdl6–1*. The apparent opposite phenotypes of *wdl5–2* and *wdl6–1* in the mechanical stress response might be a manifestation of different alleles of functionally common genes. Generation and characterization of null mutants of *WDL5* and *WDL6* by genome editing are required to clarify their functional relationships. The role and relevance of cytoskeleton in pollen germination and tube growth have been intensively studied.^18–21^ There are a lot of evidence showing that actin microfilaments involve various signaling pathways with a large number of actin-binding proteins and play an essential role in pollen tube growth.^22–24^ On the other hand, because disruption of microtubules affects only the direction of the tube growth but has no effect on its growth rate, pollen microtubules seem to be non-essential.^18,19^ However, the emerging roles of microtubules in vesicle trafficking and cell wall construction in pollen tubes have suggested the significance of microtubules in the regulation of tip growth and in the pollen tube-pistil interaction.^25–27^ The involvement of other members of the WVD2/WDL family in pollen tube growth is not known and should be investigated further.
